# Editorial: Community Engagement Is Key to Promoting Asian American, Native Hawaiian, and Pacific Islander Health

**DOI:** 10.1089/heq.2022.0124

**Published:** 2022-09-01

**Authors:** Zhuo Chen, Grace X. Ma, Xinzhi Zhang, Monica R. McLemore

**Affiliations:** ^1^ Department of Health Policy and Management, College of Public Health, University of Georgia, Athens, Georgia, USA.; ^2^ Center for Asian Health, Department of Urban Health and Population Science at Lewis Katz School of Medicine and Fox Chase Cancer Center, Temple University, Philadelphia, Pennsylvania, USA.; ^3^ Center for Translation Research & Implementation Science, National Heart, Lung, and Blood Institute, National Institutes of Health, Bethesda, Maryland, USA.; ^4^ Family Health Care Nursing Department, University of California San Francisco, San Francisco, California, USA.

The COVID-19 pandemic has inflicted immense physical and emotional pain and suffering across the board, but many minoritized and marginalized communities are hit harder than others.^1,2^ Among them, Asian American, Native Hawaiian, and Pacific Islander (AA and NH/PI) communities not only bear a disproportional morbidity and mortality burden,^3–5^ but also are the target of anti-Asian racism and hate crimes.^6–9^ Studies have identified a strong effect of job loss associated with the pandemic on psychological distress among Asian Americans and African Americans,^10^ higher ratios of COVID-19 death rates among non-Hispanic Asian Americans and Pacific Islanders relative to non-Hispanic Whites,^1^ and lower rates of telehealth utilization during the pandemic among East and Southeast Asians.^11^

With support from the Rockefeller-endowed China Medical Board, an independent American foundation, and the Blue Shield of California Foundation, *Health Equity* journal called for submissions to a special issue dedicated to research and perspectives on AA and NH/PI health, with a focus on the impact of the COVID-19 pandemic.^12,13^ We have had an overwhelming response to the call, with the final collection including 12 articles covering a wide range of topics. A brief summary of the articles is included hereunder.

The collection includes insights and perspectives on AA and NH/PI health from government, providers, and researchers. Ka'ai and her colleagues from the White House Initiative on Asian Americans, Native Hawaiians, and Pacific Islanders laid out three key health-related policy priorities of the Initiative, that is, Anti-Asian hate and violence, data disaggregation, and language access.^14^ Lee et al described how Asian Health Services, a federally qualified health center, collected and used descriptive data of their patient population to inform the rapid adoption of telehealth in response to the COVID-19 pandemic.^15^

Kaholokula et al pointed out that AA and NH/PI communities are among the racial and ethnic minority communities of priority to the NIH Community Engagement Alliance (CEAL) Against the COVID-19 Disparities, have had among the highest rates of COVID-19 incidence, hospitalizations, and deaths, and emphasized the importance of data dissagregation, culturally and linguistically appropriate intervention, workforce development, and leadership representation.^16^

AA and NH/PI communities have experienced health disparities before the COVID-19 pandemic. In this collection, Deutsch-Feldman et al used data from the National Tuberculosis Surveillance System to examine tuberculosis (TB) reported among NH/PI and found higher TB incidence among NH/PI persons, particularly those born in the US Affiliated Pacific Islands.^17^ Yin et al highlighted the higher mental health needs among Chinese Americans with Limited English Proficiency (LEP) and reviewed the effectiveness of the Chinese-language Patient Health Questionnaire 9.^18^

The COVID-19 pandemic has imposed a disproportional burden on the AA and NH/PI communities. Islam et al analyzed data from the COVID-19 Household Impact Survey and identified disparities in food insecurity and financial hardship among AA adults, in particular among Filipino and Vietnamese communities, and increased self-reported anxiety and hopelessness.^19^ Camacho and coauthors analyzed data from the Current Population Survey and found that Native Hawaiians and Pacific Islanders in the state of Washington had a higher incidence rate of unemployment claims compared with other racial groups statewide.^20^

Using data from the Health, Ethnicity, and Pandemic (HEAP) Survey, Su et al conducted a mixed method study to examine the perceived racisms among minority populations.^21^ They estimated about 19% of non-Hispanic Asian and Black people reported experiencing racial discrimination during the COVID-19 pandemic and concluded that a host of factors at the individual, household, and neighborhood levels affect the odds of perceived racism.^21^ Tiwari and Zhang identified differences in mental health status among Asian American subgroups, with South Asian Americans having significantly higher odds of experiencing psychological distress than non-Hispanic White Americans after controlling for confounders.^22^

COVID-19 vaccines are a powerful tool to contain and control the COVID-19 pandemic, but disparities in access and uptake of COVID-19 vaccines exist. Using data from the Household Pulse Survey collected in 2021, Zhang et al found that Asian Americans have the highest rate of COVID-19 vaccination, but age, gender, and education gradients exist.^23^ The authors also cautioned on the vaccination hesitation among subgroups of Asian Americans.^23^

Nguyen et al conducted a community-based qualitative study using focus groups and key informant interviews among a Vietnamese American community in Houston, Texas and concluded that promoting science-based information through trusted messengers, improving awareness and access, and showcasing benefits to the community could increase the uptake of COVID-19 vaccination among Vietnamese Americans.^24^ Samoa et al used data from the AA and NH/PI COVID-19 Needs Assessment Project and found that vaccine hesitancy ranged from 23% among NH/PIs to 57% among Tongans, with consistent associations between socioeconomic status and vaccine hesitancy among Native Hawaiians, Samoans, and Multi-ethnic NH/PIs.^25^

The collection of articles has contributed to our understanding of the health disparities experienced by AAs and NH/PIs and pointed to the urgent need to address the disparities, as Ka'ai et al noted.^14^ First, anti-Asian hate crimes have surged after the emergence of COVID-19 and exacerbated the physical and mental suffering among AAs.^14,21^ The works highlighted by the Biden Administration in addressing anti-Asian bias, xenophobia, and harassment need to be applauded and sustained.^26^ Second, LEP among AAs has been a barrier to accessing prevention and health care services,^18^ including mental health and genetic testing.^27–29^ Language assistance will help AAs to overcome barriers that are pervasive in their encounters with the health care systems. Third, research in this collection has highlighted the heterogeneities within the AA and NH/PI communities,^17,22–25^ necessitating data disaggregation for AA and NH/PI.

Emanated from the urgent need duly noted in this collection, we conclude community engagement is critical to promoting AA and NH/PI health.^30,31^ Community engagement will increase awareness of anti-Asian hate crimes, address misinformation and disinformation, and reduce the language barriers by pairing bilingual or multilingual researchers and community organizers with those who have unmet medical or mental health needs. Coordinated efforts among research communities may reduce redundancy and increase the cost-effectiveness of dissemination and implementation efforts in the diverse AA and NH/PI communities.

We end with a call for AA and NH/PI researchers and those who have the interest and passion for promoting AA and NH/PI health to coalesce and work together, reach AA and NH/PI communities, overcome barriers including LEP and unconscious biases, appreciate the vast heterogeneities within the diverse communities, and eliminate the health disparities AA and NH/PI communities are experiencing.

## Abbreviations Used

AA: Asian American
CEAL: Community Engagement Alliance
HEAP: Health, Ethnicity, and Pandemic
LEP: Limited English Proficiency
NH/PI: Native Hawaiian and Pacific Islander
TB: tuberculosis
